# Association of Social and Demographic Factors With COVID-19 Incidence and Death Rates in the US

**DOI:** 10.1001/jamanetworkopen.2020.36462

**Published:** 2021-01-29

**Authors:** Monita Karmakar, Paula M. Lantz, Renuka Tipirneni

**Affiliations:** 1 Division of General Medicine, Department of Internal Medicine, University of Michigan, Ann Arbor; 2 Gerald R. Ford School of Public Policy, University of Michigan, Ann Arbor; 3 Department of Health Management and Policy, School of Public Health, University of Michigan, Ann Arbor; 4 Institute for Healthcare Policy and Innovation, University of Michigan, Ann Arbor

## Abstract

**Importance:**

Descriptive data have revealed significant racial/ethnic disparities in coronavirus disease 2019 (COVID-19) cases in the US, but underlying mechanisms of disparities remain unknown.

**Objective:**

To examine the association between county-level sociodemographic risk factors and US COVID-19 incidence and mortality.

**Design, Setting, and Participants:**

This cross-sectional study analyzed the association between US county-level sociodemographic risk factors and COVID-19 incidence using mixed-effects negative binomial regression, and COVID-19 mortality using zero-inflated negative binomial regression. Data on COVID-19 incidence and mortality were collected from January 20 to July 29, 2020. The association of social risk factors with weekly cumulative incidence and mortality was also examined by interacting time with the index measures, using a random intercept to account for repeated measures.

**Main Outcomes and Measures:**

Sociodemographic data from publicly available data sets, including the US Centers for Disease Control and Prevention’s Social Vulnerability Index (SVI), which includes subindices of socioeconomic status, household composition and disability, racial/ethnic minority and English language proficiency status, and housing and transportation.

**Results:**

As of July 29, 2020, there were a total of 4 289 283 COVID-19 cases and 147 074 COVID-19 deaths in the US. An increase of 0.1 point in SVI score was associated with a 14.3% increase in incidence rate (incidence rate ratio [IRR], 1.14; 95% CI, 1.13-1.16; *P* < .001) and 13.7% increase in mortality rate (IRR, 1.14; 95% CI, 1.12-1.16; *P* < .001), or an excess of 87 COVID-19 cases and 3 COVID-19 deaths per 100 000 population for a SVI score change from 0.5 to 0.6 in a midsize metropolitan county; subindices were also associated with both outcomes. A 0.1-point increase in the overall SVI was associated with a 0.9% increase in weekly cumulative increase in incidence rate (IRR, 1.01; 95% CI, 1.01-1.01; *P* < .001) and 0.5% increase in mortality rate (IRR, 1.01; 95% CI, 1.01-1.01; *P* < .001).

**Conclusions and Relevance:**

In this cross-sectional study, a wide range of sociodemographic risk factors, including socioeconomic status, racial/ethnic minority status, household composition, and environmental factors, were significantly associated with COVID-19 incidence and mortality. To address inequities in the burden of the COVID-19 pandemic, these social vulnerabilities and their root causes must be addressed.

## Key Points

**Question:**

Are population-level social factors associated with coronavirus disease 2019 (COVID-19) incidence and mortality?

**Findings:**

In this cross-sectional study including 4 289 283 COVID-19 cases and 147 074 COVID-19 deaths, county-level sociodemographic risk factors as assessed by the Social Vulnerability Index were associated with greater COVID-19 incidence and mortality.

**Meaning:**

These findings suggest that to address inequities in the burden of the COVID-19 pandemic, these sociodemographic risk factors and their root causes must be addressed.

## Teaser

This cross-sectional study examines the county-level associations of sociodemographic risk factors with coronavirus disease 2019 incidence and mortality in the US.

## Introduction

While some have referred to coronavirus disease 2019 (COVID-19) as “the great equalizer,” early reports from hard-hit areas in the US suggest that the disease has a disproportionate burden associated with the longstanding social determinants of health, including racial/ethnic and socioeconomic disparities.^1,2,3^ In Michigan, one of the first states to report COVID-19 data by race/ethnicity and demographic characteristics, African American individuals initially experienced 31% of the state’s 57 397 cases despite representing only 14% of the state’s population.^4^ Mortality rates are also higher among African American, Hispanic, and Native American individuals with COVID-19.^3,5,6,7^ In New York, New York, the early epicenter of the US COVID-19 outbreak, rates of hospitalizations and deaths were highest in the Bronx, the borough with the highest proportion of members of racial/ethnic minority groups and households living in poverty.^8^

Emerging data on COVID-19 disparities are concerning. What underlying factors can explain this inequitable burden of the pandemic on low-income and minority communities? A myriad of factors have been posited, from biological, to medical risk factors, such as diabetes and lung disease, to social risk factors, such as low socioeconomic status, crowded housing, and necessary use of public transportation.^3,9^ As states and localities vary in whether and how frequently they report COVID-19 data by race/ethnicity and other sociodemographic characteristics,^1,2,3,5,8,9^ our knowledge of risk factors at the individual level is mostly limited to anecdotal reports and ecological studies.

Prior to the outbreak of severe acute respiratory syndrome coronavirus 2 (SARS-CoV-2), individuals from racial/ethnic minority groups in the US were already more likely to live in areas with significant social disadvantage, characterized by high unemployment and poverty rates, unaffordable housing, and poor health care infrastructure, which may explain the higher disease burden in these communities. Data on these types of social risks or vulnerabilities are readily available and may be examined at the level of small geographic areas, such as counties or census tracts.

Researchers have begun to explore the association between sociodemographic risk factors at the individual and community levels and COVID-19 incidence.^10,11^ Our study objective was to extend this work by assessing which sociodemographic risk factors were associated with COVID-19 outcomes over time.

## Methods

In this cross-sectional study, we investigated the association between county-level social risk factors and COVID-19 cases and deaths, as well as weekly changes in cumulative incidence and mortality, using publicly available data sets as of July 29, 2020. As a start date, we used January 20, 2020, the date of the first documented case of COVID-19 in the US. The University of Michigan institutional review board deemed the study not regulated, as it involved only analysis of publicly available deidentified data and therefore exempt from informed consent. We followed the Strengthening the Reporting of Observational Studies in Epidemiology (STROBE) reporting guideline. eTable 1 in the Supplement details each data element and source.

### COVID-19 Incidence and Mortality

Weekly cumulative COVID-19 cases and deaths in US counties were obtained from the Johns Hopkins University Center for Systems Science and Engineering data repository.^12^ We included data from March 25 to July 29, 2020 (weeks 9 to 27 of the pandemic), for our weekly time trend models to avoid inconsistencies in early reporting. The data were restricted to the 50 US states and Washington, District of Columbia. US territories, 52 observations with unassigned counties, and the 5 counties of New York City were excluded from analysis, as only aggregated data for New York City as a whole were reported.

### Social Vulnerability Index

The Social Vulnerability Index (SVI) was developed by the Centers for Disease Control and Prevention to provide a composite measure of community susceptibility to adversities in the face of health shocks, including disease outbreaks.^13^ The SVI is comprised of 4 subindices created using American Community Survey data from 2014 to 2018 on socioeconomic status (including poverty rate, unemployment rate, per capita income, and educational attainment); household composition and disability (including percentages of persons aged ≥65 years or ≤17 years, civilian noninstitutionalized population with disability, and single parent households with children aged <18 years); racial/ethnic minority status and language (includes percentages of individuals who are members of racial/ethnic minority groups [Hispanic, African American, American Indian or Alaska Native, Asian, Native Hawaiian or other Pacific Islander, or other race], and with limited English-speaking ability); and housing type and transportation (including percentages of housing in structures with 10 or more units, mobile homes, occupied housing units with more people than rooms, households without vehicles, and persons in institutionalized group quarters). Each subindex is a percentile rank. The overall SVI is calculated by adding up individual indices and converting the summated score into a percentile rank, ranging from 0 to 1, with higher values indicating greater vulnerability to a natural disaster; we rescaled by multiplying by 10 to aid interpretation. Thus, reporting of results of a 1-unit change refer to 0.1 on the original scale.

### Other Social Risk Factors

Other county-level sociodemographic measures not included in the SVI were obtained from 2014 to 2018 American Community Survey 5-year estimates. These included more detailed race/ethnicity data (percentages of individuals who are African American, Hispanic/Latinx, American Indian/Alaskan Native, or Asian); percentage of persons younger than 65 years without health insurance, percentage of individuals 16 years or older using public transport for work commuting, and the Gini index of income inequality (range from 0, indicating complete equality, to 1, indicating complete inequality). Data on food insecurity, defined as the percentage lacking access to a reliable source of food, were obtained from the Robert Wood Johnson Foundation’s 2020 county health rankings.^14^

### Population Health and Health Care Covariates

County-level numbers of primary care physicians and hospital beds were obtained from the Health Resources and Services Administration’s Area Health Resources File.^15^ Number of intensive care unit beds was obtained from the Henry J. Kaiser Family Foundation.^16^ County-level mean life expectancy and percentage of the adult population (age ≥20 years) with obesity, defined as a body mass index (calculated as weight in kilograms divided by height in meters squared) greater than or equal to 30, were obtained from the Robert Wood Johnson Foundation’s 2020 county health rankings.^14^

### Population and Testing Covariates

To calculate population density, data on county area in square miles were collected from the US Census Cartographic Boundary File. Urbanicity was defined using the 2013 Rural-Urban Continuum (Beale) codes from the US Department of Agriculture.^17^ Statewide total number of COVID-19 tests performed as of July 29, 2020 was obtained from The COVID Tracking Project.^18^

### Statistical Analysis

County-level COVID-19 incidence and mortality are reported per 100 000 population. Heat maps display total incidence and mortality rate as of July 29, 2020, superimposed over SVI for each county.

Cross-sectional data on the total number of COVID-19 cases and deaths reported as of July 29, 2020, were used to estimate the association of county characteristics with incidence and mortality on this date. We used mixed-effects negative binomial regression to model the COVID-19 case count. A mixed-effects zero-inflated negative binomial regression was used to model death counts, since 29% of the counties reported zero deaths. The number of cases per 100 000 population was used to estimate excess zeros in the zero-inflated negative binomial regression that modeled mortality, with the assumption that counties with smaller incidence rates were more likely to experience no COVID-19 deaths. All cross-sectional models included a random intercept for state. Initial bivariate analyses estimated if incidence and mortality were associated with population density, urbanicity, and COVID-19 testing rate. In subsequent analyses, each individual county characteristic was included in separate regression models to avoid potential collinearity issues.

Subsequently, serial cross-sectional data on the total number of COVID-19 cases and deaths, reported weekly between March 25 to July 29, 2020, were used to estimate the association between county SVI and weekly change in incidence and death rates. Similar to the single cross-sectional models, we used a mixed-effects negative binomial regression to model changes in weekly COVID-19 cases and a mixed-effects zero-inflated negative binomial regression to model weekly changes in COVID-19 deaths. Serial cross-sectional models included a random intercept accounting for county-level repeated measures and an interaction between centered time (in weeks) and SVI (overall or subindices) to assess whether sociodemographic risk factors were associated with weekly changes in incidence and mortality in a separate regression for each index.

Incidence rate ratios (IRRs) and estimated probabilities were used to interpret associations. All models controlled for population density, urbanicity, and COVID-19 testing rate, based on a priori conceptual framework and initial bivariate analyses.

We conducted sensitivity analyses excluding data from counties in the top 5 states with the highest COVID-19 incidence rate as of July 29, 2020. For serial cross-sectional models, we checked for robustness by adding state fixed effects to regressions.

Analyses were performed using R statistical software (glmmTMB^19^ and ggeffects^20^ packages) version 3.6.2 (R Project for Statistical Computing). Bonferroni adjustment was applied for multiple comparisons, considering 2-sided *P* < .001 statistically significant.

## Results

As of July 29, 2020, there were 4 289 283 COVID-19 cases and 147 074 deaths due to COVID-19 in 3137 US counties. The top 5 states with highest incidence rates were Arizona, Connecticut, Delaware, the District of Columbia, and Rhode Island.

**Figure 1.**
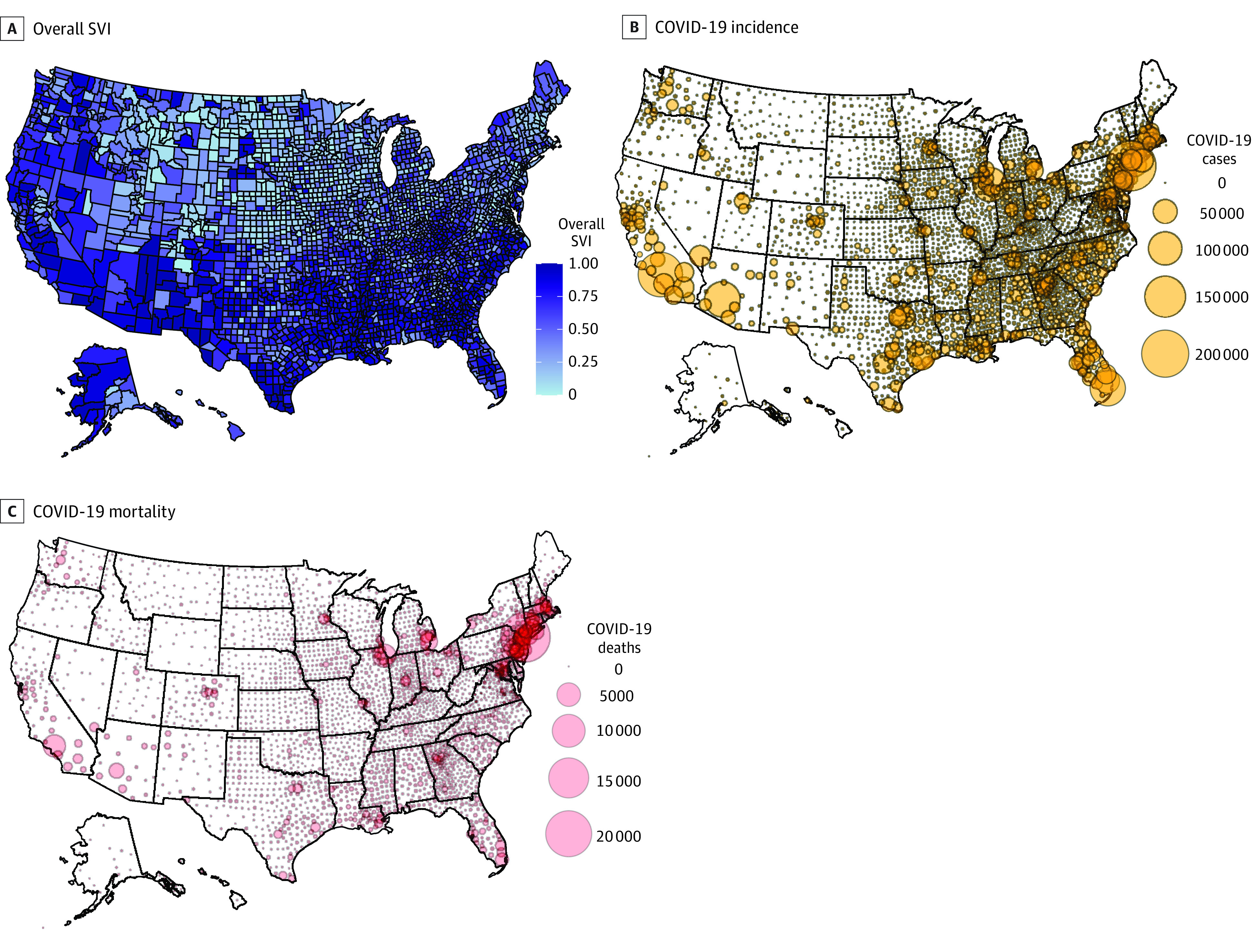
Heat Map of US Counties Showing Coronavirus Disease 2019 (COVID-19) Incidence and Mortality by Social Vulnerability Index (SVI) Higher SVI score (range, 0-1) indicates greater socioeconomic disadvantage.

Figure 1 shows a US heat map with the color gradient representing the overall SVI overlaid with COVID-19 incidence rate and death rate for each county. County-level incidence and mortality rates showed a different pattern than state rankings. The county with the highest incidence rate in the country, Trousdale County, Tennessee (16 348 cases per 100 000 population), had an overall SVI score of 0.52, and the county with the highest death rate, Hancock County, Georgia (398 deaths per 100 000 population) had an overall SVI score of 0.80 (eTable 2 in the Supplement).

In initial bivariate analyses, demographic factors (population density and urbanicity), but not COVID-19 testing rate, were significantly associated with COVID-19 incidence and mortality (eTable 3 in the Supplement). All subsequent analyses adjusted for these 3 covariates per our a priori conceptual framework.

### SVI and COVID-19 Incidence and Mortality Rates

In cross-sectional analysis, we found a significant association between SVI (overall and subindices) and COVID-19 incidence and mortality (Table 1). A 0.1-point increase in the overall SVI score was associated with a 14.3% increase in incidence rate (IRR, 1.14; 95% CI, 1.13-1.16; *P* < .001) and 13.7% increase in mortality rate (IRR, 1.14; 95% CI, 1.12-1.16; *P* < .001). In subindices analyses, racial/ethnic minority status and limited English proficiency was associated with a 21.7% increase in incidence rate per 0.1-point increase (IRR, 1.22; 95% CI, 1.20-1.23; *P* < .001), and 16.9% increase in mortality rate per 0.1-point increase (IRR, 1.17; 95% CI, 1.14-1.19; *P* < .001).

**Table 1.** Association of Social Vulnerability Index Measures With COVID-19 Incidence and Mortality

Variable	COVID-19 incidence rate^a^^,^^b^		COVID-19 mortality rate^a^^,^^c^	
	IRR (95% CI)	*P* value	IRR (95% CI)	*P* value
Overall Social Vulnerability Index^d^	1.14 (1.13-1.16)	<.001	1.14 (1.12-1.16)	<.001
Socioeconomic status subindex^d^	1.11 (1.10-1.13)	<.001	1.12 (1.09-1.14)	<.001
Household characteristics and disability subindex^d^	1.02 (1.01-1.03)	.001	1.06 (1.04-1.08)	<.001
Racial/ethnic minority status and language subindex^d^	1.22 (1.20-1.23)	<.001	1.17 (1.14-1.19)	<.001
Housing type and transportation subindex^d^	1.10 (1.08-1.11)	<.001	1.08 (1.06-1.10)	<.001

Abbreviation: COVID-19, coronavirus disease 2019; IRR, incidence rate ratio.

^a^ Each of the independent variables was entered into a separate regression model to test the association with either COVID-19 incidence or mortality, adjusted for population density, urbanicity, and COVID-19 testing rate. Each cell represents a separate regression model. All regression models included an offset for the total number of people residing in the county. Analytic sample excluded the counties spanning New York, New York.

^b^ Incidence rates were estimated using mixed-effects negative binomial regression with a random intercept for state.

^c^ Mortality rates were estimated using mixed-effects zero-inflated negative binomial regression with a random intercept for state. Cases per 100 000 population were used to model the logit part in each model estimating excess zero count.

^d^ Owing to rescaling of the variables, IRR for each index shows the change in incidence or mortality for a 0.1 unit of the original index measure.

Counties with greater SVI scores, or greater sociodemographic disadvantage, had higher COVID-19 incidence and mortality rates. For example, a midsize metropolitan county with SVI score of 0.5 (the percentile mean) was estimated to have 604 cases and 15 deaths per 100 000 population, while a county with the same characteristics but SVI score of 0.6 was estimated to have 691 cases and 18 deaths per 100 000 population. This equates to approximately 87 excess COVID-19 cases and 3 excess deaths per 100 000 for the 0.1-increase in SVI score.

### Association of SVI With Weekly Change in Cumulative COVID-19 Incidence and Mortality

In serial cross-sectional analyses, we found significant associations between SVI (overall and all 4 subindices) and weekly cumulative change in COVID-19 incidence and mortality (Table 2). A 0.1-point increase in the overall SVI score was associated with a 0.9% increase in weekly cumulative increase in incidence rate (IRR, 1.01; 95% CI, 1.01-1.01; *P* < .001) and 0.5% increase in mortality rate (IRR, 1.0; 95% CI, 1.01-1.01; *P* < .001). As in cross-sectional analysis, the racial/ethnic minority status and limited English proficiency subindex was associated with change in weekly cumulative mortality (IRR, 1.01; 95% CI, 1.01-1.01; *P* < .001), while racial/ethnic minority status and limited English proficiency (IRR, 1.01; 95% CI, 1.01-1.01, *P* < .001) and socioeconomic status (IRR, 1.01; 95% CI, 1.01-1.01, *P* < .001) were associated with change in weekly cumulative incidence. Although the IRRs may appear to be low, the scale of growth in weekly cumulative incidences is logarithmic, so the excess cases and deaths in higher vs lower SVI counties increase exponentially over time. The higher the SVI, the steeper the slope of the growth curves for COVID-19 cases and deaths.

**Table 2.** Association of Social Vulnerability Index Measures With Change in Weekly Cumulative COVID-19 Incidence and Mortality Rates

Variable	COVID-19 incidence rate^a^^,^^b^		COVID-19 mortality rate^a^^,^^c^	
	IRR (95%CI)	*P* value	IRR (95%CI)	*P* value
Overall SVI				
Time, per wk	1.26 (1.25-1.26)	<.001	1.20 (1.19-1.20)	<.001
Overall SVI index^d^	1.12 (1.10-1.13)	<.001	1.22 (1.19-1.26)	<.001
Interaction of time and overall SVI score	1.01 (1.01-1.01)	<.001	1.01 (1.01-1.01)	<.001
Socioeconomic status subindex				
Time, wk	1.26 (1.26-1.27)	<.001	1.21 (1.20-1.21)	<.001
Socioeconomic status index^d^	1.09 (1.07-1.11)	<.001	1.19 (1.16-1.22)	<.001
Interaction of time and socio-economic status index^d^	1.01 (1.01-1.01)	<.001	1.00 (1.00-1.00)	<.001
Household and disability subindex				
Time, wk	1.28 (1.27-1.28)	<.001	1.22 (1.21-1.22)	<.001
Household characteristics and disability index^d^	1.04 (1.02-1.05)	<.001	1.11 (1.08-1.14)	<.001
Interaction of time and household characteristics and disability index^d^	1.01 (1.01-1.01)	<.001	1.00 (1.00-1.00)	<.001
Racial/ethnic minority status and language subindex				
Time, wk	1.27 (1.26-1.27)	<.001	1.16 (1.16-1.17)	<.001
Racial/ethnic minority status and language index^d^	1.15 (1.13-1.17)	<.001	1.21 (1.18-1.25)	<.001
Interaction of time and racial/ethnic minority status and language index^d^	1.01 (1.01-1.01)	<.001	1.01 (1.01-1.01)	<.001
Housing and transportation sub-index				
Time, wk	1.28 (1.276-1.284)	<.001	1.20 (1.19-1.21)	<.001
Housing type and transportation index^d^	1.09 (1.08-1.11)	<.001	1.16 (1.13-1.19)	<.001
Interaction of time and housing type and transportation index^d^	1.01 (1.01-1.01)	<.001	1.01 (1.00-1.01)	<.001

Abbreviations: COVID-19, coronavirus disease 2019; IRR, incidence rate ratio.

^a^ Each of the regression models was adjusted for population density, urbanicity, and state-wide COVID-19 testing rate. Time (in weeks) was centered at week 19 (May 28, 2020–June 3, 2020). In separate regression models for each measure, time was interacted with the overall SVI score and subindices along with the individual outcomes to study how the SVI index was associated with the weekly change in COVID-19 incidence or mortality. All regression models included an offset for the total population in the county. Analytic sample exclude the counties spanning New York, New York.

^b^ Incidence rates were estimated using mixed-effects negative binomial regression with a random intercept for repeated measures within county.

^c^ Mortality rates were estimated using mixed-effects zero-inflated negative binomial regression with a random intercept for repeated measures within county. COVID-19 cases per 100 000 population were used to model the logit part in each model estimating excess zero count.

^d^ Owing to rescaling of the variables, IRR for each index shows the change in incidence or mortality for a 0.1 unit of the original index measure.

**Figure 2.**
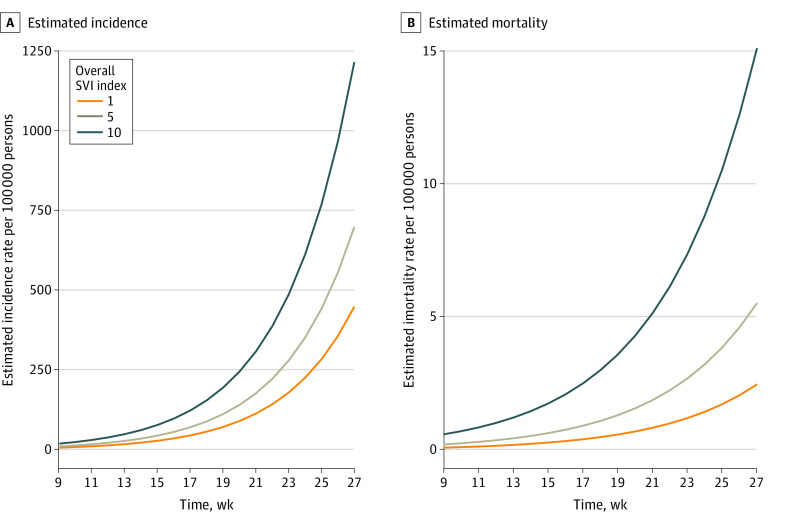
Association of Social Vulnerability Index (SVI) Values With Weekly Cumulative Coronavirus Disease 2019 Incidence and Mortality Rates Incidence and mortality are presented per 100 000 population from March 25 to July 29, 2020, in a county with a Rural-Urban Continuum Code mean of 6, defined as urban population of 2500 to 19 999, adjacent to a metropolitan area. Graphs present the estimated incidence and mortality per 100 000 with population density and state-wide COVID-19 testing rate fixed at their means, and county random effect fixed at 0. For estimated mortality, the deaths per 100 000 was fixed at the mean. Solid lines show the association between time and cumulative COVID-19 incidence and mortality. Analytic sample excluded the counties spanning New York, New York.

We also plotted estimated weekly cumulative incidence and mortality for a midsize metropolitan county and overall SVI score of either 0.1, 0.5, or 1.0 to investigate how differing levels of sociodemographic disadvantage were associated with different COVID-19 growth curves (Figure 2). The graph shows that counties with higher SVI scores had greater rates of increase in weekly cumulative incidence and mortality rates.

### Individual Sociodemographic Characteristics and COVID-19 Incidence and Mortality Rate

We also assessed the cross-sectional association of individual sociodemographic measures with COVID-19 incidence and mortality (Table 3). Most sociodemographic factors were significantly associated with both incidence and mortality. Factors associated with risk of disease incidence included percentage of the population living in crowded housing (IRR, 1.16; 95% CI, 1.15-1.18; *P* < .001), with limited English proficiency (IRR, 1.16; 95% CI, 1.14-1.17; *P* < .001), or in a single parent household (IRR, 1.11; 95% CI, 1.09-1.12; *P* < .001). Factors associated with higher mortality rate included higher percentages of the population in crowded housing (IRR, 1.15; 95% CI, 1.12-1.18; *P* < .001), in a single parent household (IRR, 1.10; 95% CI, 1.09-1.12; *P* < .001), or with limited English proficiency (IRR, 1.09; 95% CI, 1.07-1.11; *P* < .001).

**Table 3.** Association of County Sociodemographic and Health Characteristics With COVID-19 Incidence and Mortality

Characteristic	COVID-19 incidence rate^a^^,^^b^		COVID-19 mortality rate^a^^,^^c^	
	IRR (95% CI)	*P* value	IRR (95% CI)	*P* value
Socioeconomic				
Poverty rate, % under federal poverty level^d^	1.03 (1.03-1.04)	<.001	1.05 (1.04-1.05)	<.001
Unemployment rate, %^d^	1.04 (1.03-1.06)	<.001	1.07 (1.05-1.09)	<.001
Per capita income, scaled to multiple of $1000^d^	0.98 (0.97-0.98)	<.001	0.98 (0.97-0.99)	<.001
Education, % age ≥25 y with no high school degree^d^	1.07 (1.06-1.07)	<.001	1.06 (1.05-1.07)	<.001
Adults without health insurance, %	1.09 (1.08-1.10)	<.001	1.07 (1.05-1.08)	<.001
Gini income inequality index^e^	1.03 (1.02-1.04)	<.001	1.05 (1.04-1.07)	<.001
Household and disability characteristics, %				
Age, y				
≥65^d^	0.93 (0.93-0.94)	<.001	0.98 (0.97-0.99)	<.001
≤17^d^	1.07 (1.06-1.08)	<.001	1.05 (1.04-1.07)	<.001
Single parent household^d^	1.11 (1.09-1.12)	<.001	1.10 (1.09-1.12)	<.001
People with disability (noninstitutionalized)^d^	0.96 (0.96-0.97)	<.001	0.99 (0.98-1.01)	.35
Racial/ethnic minority status and language, %				
Any racial/ethnic minority^d^	1.03 (1.02-1.03)	<.001	1.03 (1.02-1.03)	<.001
African American	1.02 (1.02-1.02)	<.001	1.02 (1.02-1.03)	<.001
Hispanic or Latinx	1.02 (1.01-1.02)	<.001	1.02 (1.01-1.02)	<.001
American Indian or Alaskan Native	1.01 (1.01-1.02)	<.001	1.02 (1.02-1.03)	<.001
Asian	1.03 (1.01-1.05)	<.001	1.01 (0.99-1.03)	.37
Limited English proficiency^d^	1.16 (1.14-1.17)	<.001	1.09 (1.07-1.11)	<.001
Housing, transportation, and food, %				
Housing in structures with ≥10 units^d^	1.02 (1.02-1.03)	<.001	1.01 (1.00-1.02)	.17
Mobile homes^d^	1.01 (1.00-1.01)	.01	1.01 (1.00-1.02)	.003
Occupied housing units people > rooms^d^	1.16 (1.15-1.18)	<.001	1.15 (1.12-1.18)	<.001
Households without vehicle^d^	1.02 (1.01-1.03)	<.001	1.01 (1.00-1.02)	.21
People in institutionalized group residences^d^	1.04 (1.03-1.05)	<.001	1.08 (1.07-1.10)	<.001
Workers age ≥16 y using public transport to commute	1.05 (1.03-1.07)	<.001	1.04 (1.00-1.07)	.03
People lacking access to adequate food	1.01 (1.01-1.02)	.001	1.06 (1.04-1.07)	<.001
Population health care resource availability				
Primary care physicians, No. per 10 000 population	0.99 (0.98-1.00)	.03	0.99 (0.98-1.00)	.09
Primary care clinicians other than physicians, No. per 10 000 population	1.00 (1.00-1.01)	.22	1.00 (0.99-1.01)	.85
Hospital beds, No. per 1000 population	1.00 (0.99-1.00)	.09	1.00 (0.99-1.01)	.78
Intensive care unit beds, No. per 10 000 population	1.02 (1.01-1.04)	.004	1.01 (0.99-1.03)	.34
Population health				
Life expectancy, y	0.98 (0.97-0.99)	.001	0.95 (0.93-0.97)	<.001
Population age ≥20 y with BMI >30, %	1.02 (1.01-1.02)	<.001	1.02 (1.02-1.03)	<.001

Abbreviations: BMI, body mass index (calculated as weight in kilograms divided by height in meters squared); COVID-19, coronavirus disease 2019; IRR, incidence rate ratio.

^a^ Each of the independent variables was entered into a separate regression model to test the association with either COVID-19 incidence or mortality, adjusted for population density, urbanicity, and COVID-19 testing rate. Each cell represents a separate regression model. All regression models included an offset for the total number of people residing in the county. Analytic sample excluded the counties spanning New York, New York.

^b^ Incidence rates were estimated using mixed-effects negative binomial regression with a random intercept for state.

^c^ Mortality rates were estimated using mixed-effects zero-inflated negative binomial regression with a random intercept for state. COVID-19 cases per 100 000 population were used to model the logit part in each model estimating excess zero count.

^d^ These measures were included in the Social Vulnerability Index.

^e^ The Gini coefficient or index (range 0-1) is a measure of wealth distribution among residents in a community in which higher number represents more unequal wealth distribution and hence more inequity. The variable was rescaled by multiplying with 100 such that IRR for each index shows the change in incidence or mortality for 0.01 unit of the original index measure.

Counties with higher percentages of racial/ethnic minority populations experienced a higher burden of COVID-19 incidence (IRR, 1.03; 95% CI, 1.02-1.03; *P* < .001) and mortality (IRR, 1.03; 95% CI, 1.02-1.03, *P* < .001). Examining at the data more granularly, higher incidence rates were noted in counties with higher percentages of people identifying as African American (IRR, 1.02; 95% CI, 1.02-1.02; *P* < .001), Hispanic or Latinx (IRR, 1.02; 95% CI, 1.01-1.02; *P* < .001), American Indian or Alaskan Native (IRR, 1.01; 95% CI, 1.01-1.02; *P* < .001), and Asian (IRR, 1.03; 95% CI, 1.01-1.05; *P* < .001). Higher mortality rates were also observed in counties with higher percentages of people identifying as African American (IRR, 1.02; 95% CI, 1.02-1.03; *P* < .001), Hispanic or Latinx (IRR, 1.02; 95% CI, 1.01-1.02; *P* < .001), and American Indian or Alaskan Native (IRR, 1.02; 95% CI, 1.02-1.03; *P* < .001).

### Association of Population Health and Health Care Measures With COVID-19 Incidence and Mortality Rate

Most of the population health or health care availability measures were not significantly associated with COVID-19 incidence (Table 3). Obesity rate was significantly associated with COVID-19 incidence (IRR, 1.02; 95% CI, 1.01-1.02; *P* < .001) and mortality (IRR, 1.02; 95% CI, 1.02-1.03; *P* < .001). As expected, life expectancy was significantly associated with mortality, such that an additional year of life expectancy was associated with lower mortality rates (IRR, 0.95; 95% CI, 0.93-0.97; *P* < .001).

### Sensitivity Analyses

In sensitivity analyses excluding the 5 states with the highest COVID-19 incidence rates, the overall SVI and subindices remained associated with COVID-19 incidence and mortality rates, as did most of the individual sociodemographic measures examined. Similarly, in serial cross-sectional analyses, inclusion of state fixed effects did not alter our findings.

## Discussion

In this cross-sectional study of US county-level sociodemographic risk factors conducted 6 months into the country’s SARS-CoV-2 outbreak, we found significant associations with COVID-19 incidence and mortality across all social domains. The SVI, particularly the minority status and English language proficiency subindex, and many sociodemographic factors examined were associated with incidence and mortality rates. Our analysis revealed that racial/ethnic minority status was significantly associated with COVID-19 incidence and mortality, reinforcing anecdotal evidence on disparities from the field and those preliminarily reported by state health departments.^2,3,5,6,8^ Our serial cross-sectional analysis also found that social factors were associated with the rate of increase of COVID-19 cases and deaths in US counties. This extends the work of previous cross-sectional studies that examined the association of social risk factors earlier in the COVID-19 pandemic.^10,11^

These findings suggest that a significant component of COVID-19 racial/ethnic disparities are associated with community-level social factors. That is, the racial/ethnic disparities apparent in descriptive statistics are revealing underlying disparities in myriad social factors at the macro and mezzo levels known to be associated with disparities in health outcomes, including structural racism. Communities with socioenvironmental conditions, such as crowded housing and reliance on public transportation, are at higher risk of disease transmission owing to difficulty maintaining social distancing.^21^ Residents of low-income and racial/ethnic minority communities are also more likely to have essential worker occupations, which put them at higher risk of person-to-person SARS-CoV-2 exposure and transmission.^22,23^ In addition, while certain racial/ethnic minority groups are known to have higher rates of medical risk factors for COVID-19 morbidity and mortality, such as diabetes, hypertension, and lung disease,^24,25^ prior literature indicates that it is the upstream social determinants of health that are the underlying factors associated with observed disparities in these chronic health conditions.^26^

### Policy Implications

Our county-level analysis also suggests different factors for what geographic areas are associated with higher risk for COVID-19, as the counties with the highest incidence rates were not necessarily in the states with the highest number of cases and were often rural counties with predominantly White populations. This underscores that COVID-19 is not simply a problem of people of certain races/ethnicities or socioeconomic position living in certain cities. Longstanding social inequities—and the living and working conditions they generate—are associated with many aspects of the pandemic, including its severity and timing.

Furthermore, the profound economic impact of the pandemic, including the stay-at-home orders and other public health emergency policies necessary to contain SARS-CoV-2, will likely only exacerbate inequities in the social determinants of health, including unemployment, poverty, food insecurity, poor quality education, lack of health insurance or paid sick leave, and lack of access to the internet. These social conditions, driven by public policy choices, in turn may create a vicious cycle of increasing disease transmission and perpetuation of inequities in COVID-19 outcomes if underlying social factors are not considered and addressed.

SARS-CoV-2 neither created the conditions for health disparities nor did it reveal previously unrecognized social inequality. Rather, this pandemic has exacerbated longstanding racial/ethnic, social, political, and economic inequities in the US to once again ensure that the most marginalized and underresourced communities experience the worst outcomes.^27^ The difficult but crucial task for local, regional, and national policy makers will be to address, in addition to downstream health care issues, the numerous upstream and midstream social factors associated with health and health care disparities.^27^

Our findings demonstrate that the SVI and other sociodemographic measures may be used to target counties or other small geographic areas for expanded COVID-19 testing and treatment sites, as well as identification of sociodemographic risk factors for the purpose of linkage to resources, such as housing or food assistance. As limited English proficiency was significantly associated with COVID-19 outcomes, public service announcements in other languages could be used to disseminate public health guidelines regarding mask use, social distancing, and other mitigation strategies. Local and state public health departments should also coordinate with community-based social service organizations to assess and address social needs that might contribute to SARS-CoV-2 transmission. Additionally, social welfare policies aimed at increasing income, housing, and food security are essential for addressing COVID-19 along with other health disparities.

### Limitations

Our study has several limitations. First, our county-level analysis investigates population-level risk but may obscure important individual-level risk factors, including social and medical risks. Second, disease incidence is dependent on testing, and to our knowledge, there is no definitive research on whether disparities in access to testing exist in the US. We adjusted for state-level testing rates, but nationwide county-level data on testing were not available for analysis. Third, our assessment of mortality is dependent on accurate reporting and likely underestimates the full number of COVID-19–related deaths.^5,28,29,30^ If underresourced counties with greater social risk factors had lower access to testing and thus fewer deaths attributed to COVID-19, this would likely bias our results to the null. Fourth, our analysis only captures and thus represents the first 6 months of the COVID-19 pandemic in the US. Longer-term analysis might reveal different findings regarding the association between COVID-19 outcomes and county-level social factors. Fifth, we were not able to incorporate into our study design factors that may have influenced early COVID-19 spread in the US, such as international travel, although these factors were likely less relevant at the time of our analysis 6 months into the US pandemic. Sixth, some early reports suggested that essential workers, such as health care, emergency services, law enforcement, grocery store, transportation, and other workers, were at a higher risk of COVID-19.^23^ We did not incorporate this important labor market consideration, as it was out of scope for our analysis of county-level social risk factors. Additionally, we focused our analysis at the county level owing to data availability, but important variation in social risks exists within counties. Future research should examine smaller geographic areas to more closely identify communities with greater social vulnerability and risk of COVID-19. Furthermore, we focused on disease incidence and mortality, but the association of social factors with hospitalizations and other outcomes are worthy of examination.^31,32^

## Conclusions

This cross-sectional study found that a wide range of social factors, including socioeconomic status, racial/ethnic minority status, family or household composition, and environmental factors, were significantly associated with COVID-19 incidence and mortality, which are also largely considered the driving forces associated with the racial/ethnic and social disparities that are already apparent in the COVID-19 pandemic in the US. To truly bend the curve of disparities in COVID-19 and any future epidemics or pandemics, these social risk factors and their root causes must be addressed through bold policy action and societal investment.

## Notes

Supplement. **eTable 1.** Variable List and Data Sources **eTable 2.** Top 5 Counties with Highest COVID-19 Incidence and Mortality Rates and Corresponding Social Vulnerability Index Scores **eTable 3.** Association of County-level Demographic and State-level Policy Factors with COVID-19 Incidence and Mortality Rates in 50 US States and the District of Columbia
