# Supplementary material for: Association of Social and Demographic Factors With COVID-19 Incidence and Death Rates in the US
Source: JAMA Netw Open. 2021 Jan 29;4(1):e2036462. doi: 10.1001/jamanetworkopen.2020.36462 (PMC7846939; doi:10.1001/jamanetworkopen.2020.36462)
Supplement: Supplement. — eTable 1. Variable List and Data Sources eTable 2. Top 5 Counties with Highest COVID-19 Incidence and Mortality Rates and Corresponding Social Vulnerability Index Scores eTable 3. Association of County-level Demographic and State-level Policy Factors with COVID-19 Incidence and Mortality Rates in 50 US States and the District of Columbia [file jamanetwopen-e2036462-s001.pdf]

Supplemental Online Content

Karmakar M, Lantz PM, Tipirneni R. Association of social and demographic factors with COVID-19 incidence and death rates in the US. *JAMA Netw Open*. 2021;4(1):e2036462. doi:10.1001/jamanetworkopen.2020.36462

eTable 1. Variable List and Data Sources
eTable 2. Top 5 Counties with Highest COVID-19 Incidence and Mortality Rates and Corresponding Social Vulnerability Index Scores
eTable 3. Association of County-level Demographic and State-level Policy Factors with COVID-19 Incidence and Mortality Rates in 50 US States and the District of Columbia

This supplemental material has been provided by the authors to give readers additional information about their work.

© 2021 Karmakar M et al. *JAMA Network Open.*

eTable 1. Variable List and Data Sources

Variable	Variable detail	Original data source	Data source used for study[a]
**Population density**			
Total population	Population estimate	2014-2018 American Community Survey	Centers for Disease Control and Prevention (CDC): https://svi.cdc.gov/data-and-tools-download.html
Geographic area	Tract area in square miles	United States (US) Census Cartographic Boundary File - U.S. Tracts 2018 at 1:500,000 resolution	CDC: https://svi.cdc.gov/data-and-tools-download.html
**Social Vulnerability Index and component measures**			
Overall Social Vulnerability Index	Social Vulnerability Index	Centers for Disease Control and Prevention	CDC: https://svi.cdc.gov/data-and-tools-download.html
Socioeconomic status index	Sub-domain index of Social Vulnerability Index that includes component measures below	Centers for Disease Control and Prevention	CDC: https://svi.cdc.gov/data-and-tools-download.html
Poverty rate	Percentage of persons below US poverty level	2014-2018 American Community Survey	CDC: https://svi.cdc.gov/data-and-tools-download.html
Unemployment rate	Percentage unemployed	2014-2018 American Community Survey	CDC: https://svi.cdc.gov/data-and-tools-download.html
Income	Per capita income	2014-2018 American Community Survey	CDC: https://svi.cdc.gov/data-and-tools-download.html
Educational attainment	Percentage of persons with no high school diploma (age 25+) estimate	2014-2018 American Community Survey	CDC: https://svi.cdc.gov/data-and-tools-download.html

© 2021 Karmakar M et al. *JAMA Network Open.*

Variable	Variable detail	Original data source	Data source used for study[a]
Household characteristics and disability index	Sub-domain index of Social Vulnerability Index that includes component measures below	Centers for Disease Control and Prevention	CDC: https://svi.cdc.gov/data-and-tools-download.html
65 years or older	Percentage of persons age 65 and older	2014-2018 American Community Survey	CDC: https://svi.cdc.gov/data-and-tools-download.html
17 years or younger	Percentage of persons age 17 and younger	2014-2018 American Community Survey	CDC: https://svi.cdc.gov/data-and-tools-download.html
Disability	Percentage of civilian noninstitutionalized population older than 5 years with a disability estimate	2014-2018 American Community Survey	CDC: https://svi.cdc.gov/data-and-tools-download.html
Single parent household	Percentage of single parent households with children under 18	2014-2018 American Community Survey	CDC: https://svi.cdc.gov/data-and-tools-download.html
Minority status and language index	Sub-domain index of Social Vulnerability Index that includes component measures below	Centers for Disease Control and Prevention	CDC: https://svi.cdc.gov/data-and-tools-download.html
Minority	Percentage minority (all persons except white, non-Hispanic)	2014-2018 American Community Survey	CDC: https://svi.cdc.gov/data-and-tools-download.html
Limited English proficiency	Percentage of persons (age 5+) who speak English "less than well"	2014-2018 American Community Survey	CDC: https://svi.cdc.gov/data-and-tools-download.html

© 2021 Karmakar M et al. *JAMA Network Open.*

Variable	Variable detail	Original data source	Data source used for study[a]
Housing type and transportation index	Sub-domain index of Social Vulnerability Index that includes component measures below	Centers for Disease Control and Prevention	CDC: https://svi.cdc.gov/data-and-tools-download.html
Multi-unit structure	Percentage of housing in structures with 10 or more units	2014-2018 American Community Survey	CDC: https://svi.cdc.gov/data-and-tools-download.html
Mobile homes	Percentage of mobile homes	2014-2018 American Community Survey	CDC: https://svi.cdc.gov/data-and-tools-download.html
Crowding	Percentage of occupied housing units with more people than rooms	2014-2018 American Community Survey	CDC: https://svi.cdc.gov/data-and-tools-download.html
No vehicle	Percentage of households with no vehicle available	2014-2018 American Community Survey	CDC: https://svi.cdc.gov/data-and-tools-download.html
Group quarters	Percentage of persons in institutionalized group quarters	2014-2018 American Community Survey	CDC: https://svi.cdc.gov/data-and-tools-download.html
**Other sociodemographic characteristics**			
Income inequality	Gini index of income inequality	2018 American Community Survey	US Census: https://data.census.gov/cedsci/
Health insurance coverage	% under age 65 without health insurance	2014-2018 American Community Survey	US Census: https://data.census.gov/cedsci/
Public transportation to commute to work	% workers age 16 or older using public transport (excluding taxicab) to commute to work	2014-2018 American Community Survey	US Census: https://data.census.gov/cedsci/
Urbanicity	Rural-Urban Continuum (Beale) Codes	2013 Rural-Urban Continuum Codes from US Department of Agriculture	US Department of Agriculture: https://www.ers.usda.gov/data-products/rural-urban-continuum-codes.aspx

© 2021 Karmakar M et al. *JAMA Network Open.*

Variable	Variable detail	Original data source	Data source used for study[a]
Food insecurity	Percentage of population who lack adequate access to food	Map the Meal Gap from Feeding America 2017	Robert Wood Johnson Foundation and University of Wisconsin Population Health Institute 2020 County Health Rankings: https://www.countyhealthrankings.org/
**Population health care resources**			
Total primary care physicians	Total primary care physicians	2017 American Medical Association Masterfile	Health Resources and Services Administration's Area Health Resources File: https://data.hrsa.gov/topics/health-workforce/ahrf
Total hospital beds	Total hospital beds	2017 American Hospital Association Survey Database	Health Resources and Services Administration's Area Health Resources File: https://data.hrsa.gov/topics/health-workforce/ahrf
Total intensive care unit (ICU) beds	Total ICU beds	Henry J. Kaiser Family Foundation	Kaiser Family Foundation: https://khn.org/news/as,coronavirus,spreads,widely,millions,of,older,americans,live,in,counties,with,no,icu,beds/#lookup
Total other primary care providers	Total number of primary care providers other than physicians	Centers for Medicare and Medicaid Services, National Provider Identification, 2019	Robert Wood Johnson Foundation and University of Wisconsin Population Health Institute 2020 County Health Rankings: https://www.countyhealthrankings.org/

© 2021 Karmakar M et al. *JAMA Network Open.*

**Variable**	**Variable detail**	**Original data source**	**Data source used for study**[a]
**Population health measures**			
Life expectancy	Average number of years a person can expect to live	National Center for Health Statistics - Mortality Files 2016-2018	Robert Wood Johnson Foundation and University of Wisconsin Population Health Institute 2020 County Health Rankings: https://www.countyhealthrankings.org/
Obesity	Percentage of the adult population (age 20 and older) that reports a body mass index greater than or equal to 30 kg/m^2	2016 United States Diabetes Surveillance System	Robert Wood Johnson Foundation and University of Wisconsin Population Health Institute 2020 County Health Rankings: https://www.countyhealthrankings.org/

[a] Data was obtained from sources other than original data source in some cases, as indicated

© 2021 Karmakar M et al. *JAMA Network Open.*

eTable 2. Top 5 Counties with Highest COVID-19 Incidence and Mortality Rate and Corresponding Social Vulnerability Index

County[a]	State	Incidence rate	Mortality rate	Overall SVI index	Socio-economic status index	Household characteristics and disability index	Minority status and language index	Housing type and transportation index
**Counties with the highest incidence rate**								
Trousdale	Tennessee	16348.06	62.68	0.52	0.81	0.22	0.42	0.29
Lake	Tennessee	9580.12	0.00	0.92	0.98	0.61	0.39	0.95
Lee	Arkansas	9321.13	63.84	0.97	0.99	0.71	0.77	0.91
Dakota	Nebraska	9317.32	191.96	0.86	0.72	0.54	0.96	0.81
Buena Vista	Iowa	8770.98	59.23	0.74	0.58	0.41	0.93	0.71
**Counties with the highest mortality rate**								
Hancock	Georgia	3093.15	398.36	0.80	0.83	0.19	0.59	0.92
Randolph	Georgia	3315.93	366.87	0.97	0.96	0.99	0.48	0.89
Terrell	Georgia	3126.76	327.35	0.96	0.97	0.88	0.52	0.94
Early	Georgia	3150.37	299.57	0.92	0.94	0.94	0.45	0.80
McKinley	New Mexico	5400.21	296.50	0.99	1.00	0.88	0.95	0.83

[a] The counties spanning New York City were excluded from this analysis.
Abbreviations: SVI, Social Vulnerability Index.

eTable 3. Association of County-level Demographic and State-level Policy Factors with COVID-19 Incidence and Mortality Rate in 50 US States and District of Columbia

© 2021 Karmakar M et al. *JAMA Network Open.*

	Unadjusted[a]			Adjusted[b]		
	IRR	(95% CI)	*P* value	IRR	(95% CI)	*P* value
**Association with incidence rate[c]**						
County-level demographic factors						
Population density (rescaled to multiples of 100 people)	1.021	1.016 – 1.026	<0.001	1.013	1.009 – 1.018	<0.001
Urbanicity	0.930	0.920 – 0.941	<0.001	0.942	0.931 – 0.953	<0.001
State-level policy factors						
COVID-19 testing per 1000 population	1.003	0.999 – 1.006	0.13	1.002	0.998 – 1.005	0.34
**Association with mortality rate[d]**						
County-level demographic factors						
Population density (rescaled to multiples of 100 people)	1.021	1.014 – 1.027	<0.001	1.018	1.012 – 1.025	<0.001
Urbanicity	0.960	0.943 – 0.978	<0.001	0.982	0.963 – 1.001	0.06
State-level policy factors						
COVID-19 testing per 1000 population	1.005	0.999 – 1.010	0.08	1.004	0.998 – 1.009	0.17

Abbreviations: IRR, incidence rate ratio; CI, confidence interval.
[a] Each of the independent variables was entered into a separate regression model to test the bivariate association with either COVID-19 incidence or mortality. Each cell represents a separate regression model. All regression models included an offset for the total number of people residing in the county. Analytic sample excluded the counties spanning New York City.
[b] Model estimates the adjusted effect of each independent variable after all independent variables were entered into a single multivariable regression.
[c] Incidence rates were estimated using mixed effects negative binomial regression with a random intercept for state. All regression models included an offset for the total number of people residing in the county.
[d] Mortality rates were estimated using mixed effects zero inflated negative binomial regression with a random intercept for state. All regression models included an offset for the total number of people residing in the county. Cases per 100,000 population were used to model the logit part in each model predicting excess zero count.
